# CARP VIII antibody-related autoimmune cerebellar ataxia in a child after *Mycoplasma pneumoniae* infection: a case report

**DOI:** 10.3389/fimmu.2024.1480212

**Published:** 2025-01-14

**Authors:** Minglei Li, Zongming Han, Jinlei Li, Qianyun Wang, Zufang Lv

**Affiliations:** ^1^ First Department of Pediatrics, Weifang People’s Hospital Affiliated to Shandong Second Medical University, Weifang, China; ^2^ School of Clinical Medicine, Shandong Second Medical University, Weifang, China; ^3^ Third Department of Neurosurgery, Weifang People’s Hospital Affiliated to Shandong Second Medical University, Weifang, China

**Keywords:** cerebellar ataxia, autoimmune, pediatrics, *Mycoplasma pneumoniae*, carbonic anhydrase-related protein VIII (CARP VIII) antibodies

## Abstract

Autoimmune cerebellar ataxia (ACA) is a cerebellar syndrome induced by autoimmune reactions and its onset is induced by malignant tumors, prodromic infection, and gluten allergy. Its clinical symptoms include gait disorder, limb ataxia, dysarthria, and dysphagia. According to *the Chinese Expert Consensus on the Diagnosis and Management of Autoimmune Encephalitis 2024*, the diagnosis of ACA is based on the following points: 1. subacute or acute onset of the disease, with cerebellar syndrome as the main manifestation; 2. The cranial magnetic resonance imaging (MRI) in the early stage of the disease (within three months) does not show significant atrophy of the cerebellum and brainstem; 3. presence of either of the following: 1) positive anti-cerebellar antibodies in serum and/or cerebrospinal fluid cell-based assay (CBA), 2) at least two of the following are present: ① the patient or first-degree relative has a history of autoimmune disease, ② cerebrospinal fluid leukocytes >5×10^6^/L, or positive for cerebrospinal fluid specific oligoclonal bands, ③ tissue-based assay (TBA) revealing the characteristic fluorescent form of Purkinje cell antibody, and ④ the presence of systemic autoimmune disease-related antibodies; and 4. the absence of other diseases. Currently, fewer instances of ACA have been associated with positive results for carbonic anhydrase-related protein VIII (CARP VIII). Three case reports have been detected by this antibody in adults with ovarian cancer, breast cancer, or melanoma, and there is no report on this antibody in children. Moreover, neurological diseases associated with mycoplasma pneumoniae infection are increasingly being reported. Therefore, the correlation between this infection and autoimmune encephalitis antibodies needs to be further investigated.

## 1 Case report

**Figure 1:**
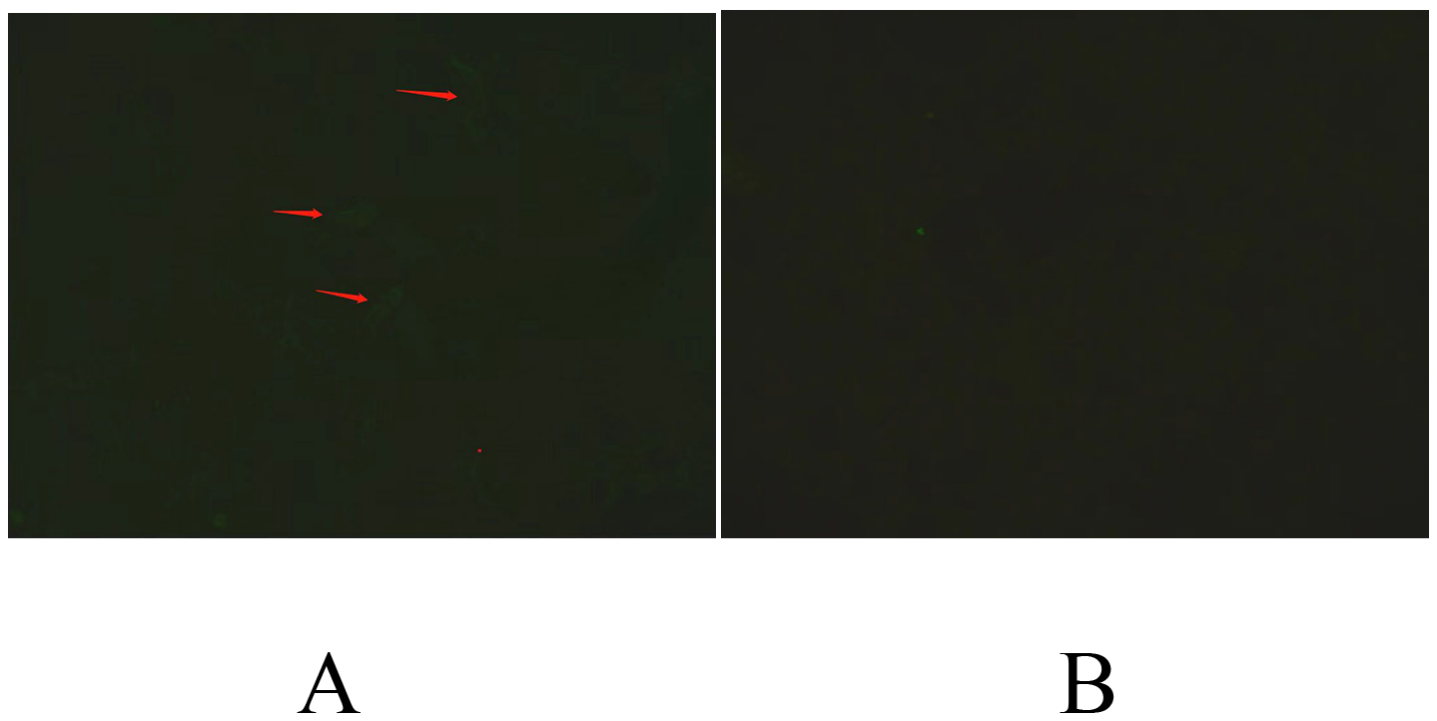
CBA (cell-based assay): Serum samples were incubated with human embryonic kidney (HEK) transfected with CARP VIII. **(A)** A specific reaction was detected when HEK cells transfected with CARP VIII were incubated with patient serum. A specific fluorescence could be seen in the cytoplasm, as indicated by the arrows, revealed a positive reaction. **(B)** On the contrary, no specific fluorescence was observed in the cytoplasm after incubation with serum from healthy people, indicating a negative reaction.

Here, we report a 9-year-old girl who was admitted due to a 20-day history of fever and cough, accompanied by unsteady gait for the past 11 days. Previously, she was diagnosed with *Mycoplasma pneumoniae* (*M. pneumoniae*) pneumonia through lung CT and serum *M. pneumoniae* antibody test at a local hospital because of fever and cough at the beginning of the disease. The patient was out on Azithromycin (10 mg/kg.d, qd × 3 d, stopped for 3 d, and qd × 3 d again) treatment. This treatment resolved the symptoms and the patient was discharged after 9 d. On the day of discharge, she presented with gait symptoms, manifested as unsteady sitting, unstable walking and slower speech, and was admitted to our hospital. Her personal and family history was unremarkable. On admission, the general physical examination revealed 1 melanocytic nevus in the right axilla, measuring about 0.2 cm × 0.3 cm (which existed since birth, and did not change significantly in recent past. After consultation with our dermatology department, the no biopsy was performed. The patient has no abnormalities in the heart, lungs and abdomen. The patient presented with a conscious state, normal vital signs, and a significantly slowed speech rate. Physical examination revealed an ataxic gait, unable to turn voluntarily, difficulty maintaining a straight line while walking, and a positive Romberg sign. Although the finger-to-nose, rotation, and heel-knee-shin tests were performed normally, there was no evidence of meningeal irritation or bilateral Babinski reflex. The following auxiliary examination were performed. General examination found no abnormalities in blood routine examination, blood biochemistry, ceruloplasmin, blood homocysteine assay, and blood ammonia assay. Abnormal serum results:antinuclear antibody spectrum including ANA (antinuclear antibody IgG type IIF) cytoplasmic fiber type 1: 320 (reactive: ≥1: 100), *M. pneumoniae* antibody (IgG/IgM) quantitative assay: MP/IgM 9.89 COI (reactive: ≥1.1);no abnormalities were found in chest CT, abdominal CT and gynecological ultrasound. Neurological examination found cerebrospinal fluid nucleated cell count 6×10^6^/L, monocytes 83%, apocytes 17%, negative tests for Pandy assay, cerebrospinal fluid protein (179.4 mg/L), cerebrospinal fluid glucose (4.0 mmol/L), cerebrospinal fluid chloride (130.3 mmol/L), normal results for cerebrospinal fluid culture and cerebrospinal fluid mycoplasma pneumoniae antibody, negative results for serum and cerebrospinal fluid autoimmune encephalitis spectrum [anti-glutamic acid decarboxylase (GAD65) antibody IgG, anti-glutamate receptor (NMDA) antibody IgG, anti-glutamate receptor (AMPA1) antibody IgG, anti-glutamate receptor (AMPA2) antibody, anti-leucine rich glioma inactivated protein 1 (LGI 1) antibody IgG, anti-contacting associated protein 2 (CASPR2) antibody IgG, GABA B receptor antibody IgG, MOG antibody IgG], negative results for oligoclonal bands and anti-AQP4 antibody IgG, serum and cerebrospinal fluid autoimmune cerebellitis spectrum [Yo/CDR2 antibody IgG, anti-DNER antibody IgG, ITPR1 antibody IgG, CARP VIII antibody IgG, PCA-2 antibody IgG, anti-glutamic acid decarboxylase (GAD65) antibody IgG] suggested serum CARP VIII antibody IgG (+) 1: 10 (**Figure 1**), and negative findings for cerebrospinal fluid (Tested by Hangzhou Oumeng WeiYi Medical Laboratory, cranial magnetic resonance imaging(MRI), cranial magnetic resonance venous imaging(MRV), cranialmagnetic resonance arterial imaging(MRA) and spinal cord magnetic resonance imaging(MRI) scan were unremarkable. Electroencephalogram(EEG) showed normal results.

Upon admission, the patient received oral doxycycline hydrochloride (2 mg/kg each time, bid × 10 d) and intravenous gamma-globulin (400 mg/kg/day for 5 days). After three days of gamma-globulin infusion, the child’s unsteady gait improved, with reduced side-to-side sway and a negative Romberg sign. However, the child remained unable to walk in a straight line. After 10 days, the child could turn around independently with normal speech, but was still unable to walk in a straight line. Therefore, methylprednisolone (20 mg/kg·d shock therapy was administered for 3 d) was applied, and CARP VIII antibody IgG in serum (-) was examined two weeks later, before the patient was discharged. The patient was prescribed to take sequential doses of oral prednisone for 6 months, together with calcium, vitamin D and potassium. After 1 month of prednisone treatment, the child returned to the hospital for a follow-up examination, and the gait was stable and she could walk in a straight line, because the child’s clinical manifestations improved, the cranial MRI scan was not repeated.

## 2 Discussion

ACA is one of the most common causes of acquired cerebellar ataxia. Depending on whether it is induced by a tumor, ACA can be categorized as paraneoplastic ACA (i.e., paraneoplastic cerebellar degeneration (PCD)) or non-paraneoplastic ACA. Non-paraneoplastic ACA include anti-glutamic acid decarboxylase (GAD) cerebellar ataxia, primary autoimmune cerebellar ataxia (PACA), and gluten ataxia (GA) (1–3).

To date, over 20 ACA-related neural antibodies have been reported. Evidence demonstrates the involvement of neural antibody in the pathogenesis of ACA and is now considered a robust diagnostic marker for ACA (4). According to *the Chinese Expert Consensus on the Diagnosis and Management of Autoimmune Encephalitis 2024*, for patients with acute or subacute cerebellar syndromes of unknown etiology, anti-neurologic antibody testing should be performed through simultaneous testing of serum and cerebrospinal fluid specimens. Anti-GAD cerebellar ataxia is based on positive cerebrospinal fluid; for other antibodies, serum and (or) positive cerebrospinal fluid anti-neurologic antibody may be used (5). Recently, several CARP VIII antibody-associated neurological disorders have been reported, and the tumors commonly associated with CARP VIII-induced ACA include melanoma, ovarian cancer, and breast cancer (6–8). CARP VIII belongs to the carbonic anhydrase (CA) family, which activates α-CA, promotes the decomposition of H_2_CO_3_ into H_2_O+CO_2_, binds to the inositol triphosphate receptor (ITPR1) of Purkinje’s fiber, and decreases the sensitivity of ITPR1 to inositol triphosphate (IP3), thereby modulating the release of Ca^2+^ from the sarcoplasmic reticulum and maintaining Ca^2+^ homeostasis in Purkinje’s fiber. Therefore, it influences the plasticity of Purkinje’s fiber. Notably, CARP VIII differs from the CA family in that it lacks zinc-binding histidine residues and hence the CA activity (9). Aspatwar et al. (10) explored the expression of CARP VIII in normal and tumor tissues. High mRNA expression of CARP VIII was been reported in the cerebellum, as well as other organs including the liver, lungs, heart, intestine, thymus and kidneys (11, 12). Elevated CARP VIII mRNA was found in several cancers. The expression of CARP VIII in the cerebellum suggests its role in brain function (9). Clinical features of patients with mutations in the CARP VIII gene include cerebellar ataxia, dysarthria, mild intellectual retardation, tremor and strabismus, the evidence for the involvement of CARP VIII gene in neurodegeneration and ataxia comes from wobbly mice, which exhibited wobbly left-right ataxic movements (10). The wobbly mice show abnormal expression of several genes involved in the synaptogenesis, synaptic vesicle formation and transport, cell proliferation and differentiation, and signal transduction. Ultrastructural abnormalities likely affect specific cerebellar cortical neurons. The extensive dysregulation of genes in wobbly mice indicates a critical physiological role for CARP VIII within the cerebellar cortex (13).

In this case, the child was initially diagnosed with acute cerebellar ataxia after admission to the hospital. No abnormality was found in the cranial MRI, and the cranial MRI was not reviewed. A study by Wang et al. showed that the cranial MRI image of a patient with ACA was normal in the initial consultation, while cerebellar atrophy appeared 5 months later (14), the severity of atrophy varied with the disease duration (1). A study by ILiat SA (15) showed that Anti-glutamic acid decarboxylase (GAD) antibody-associated cerebellar ataxia occurrs in allo-HCT, cranial MRI revealed cerebellar atrophy on brain imaging. Therefore, the imaging manifestations of this child should be followed up for at least 6 months. Studies have shown that anti-CARP VIII antibodies may be involved in the pathogenesis of various PCD, such as melanoma, ovarian cancer, and breast cancer. The exact mechanism of cerebellar degeneration is not well understood and the most probable hypothesis is that the antibodies trigger a T cell-mediated reaction but have no direct pathogenic effect (7). Romana et al. (7) reported that anti-CARP VIII antibody-mediated PCD failed to respond to chemotherapy and immunotherapy. However, the child in the present case had weakly positive anti-CARP VIII antibodies (1:10) in serum, but the antibody was not detected in the cerebrospinal fluid. After 2 weeks of anti-immunotherapy, the antibodies tests were negative. Therefore, we speculated that the pathogenesis of ACA in this child was likely to be immune-mediated after mycoplasma pneumoniae infection, and that the anti-CARP VIII antibodies served as effective markers of infection. Nevertheless, the pathogenesis of this antibody remain unknown. In this case, no tumor was found in chest, upper abdomen and pelvis CT, and no solid mass was found in gynecological ultrasound. However, a black nevus was detected in the right axilla of the child, measuring about 0.2cm*0.3cm, which existed since birth and did not changed significantly in the recent past. It was diagnosed as a pigmented nevus in dermatology, but the dermatologist did not recommend biopsy. Although no tumor was detected, dynamic follow-up is recommended, and skin biopsy should be performed to further exclude PCD when necessary.

In this case, there were typical symptoms, signs and imaging features of mycoplasma pneumoniae pneumonia were detected in the early stage. Serum mycoplasma pneumoniae antibody was positive, accompanied by acute cerebellar ataxia and elevated conventional nucleated cell counts in cerebrospinal fluid with a predominance of monocytes. Although mycoplasma pneumoniae antibody in the cerebrospinal fluid was negative, considering that it takes 1-2 weeks for the MP-IgM to enter the cerebrospinal fluid, delayed-onset mycoplasma pneumoniae encephalitis could not be excluded. This type of encephalitis is primarily caused by immune-mediated reactions, and its clinical presentation is atypical, with neurological symptoms appearing 7 days after the onset of fever (16, 17), accompanied with decreased consciousness and convulsions, and in severe cases, acute disseminated encephalomyelitis (ADEM) and other demyelinating leukoencephalopathies may occur (18). Guo QFet al. (19) explored that cortical involvement was the most common finding on neuroimaging of Mycoplasma pneumoniae encephalitis, some patients presented reversible lesions involving the splenium of the corpus callosum, which were categorized as reversible splenial lesion syndrome (RESLES),cerebellar involvement occurred in 3.4% of patients. In this case, the child’s symptoms improved after treatment with glucocorticoids and immunoglobulin, possibly due to the ACA induced by mycoplasma pneumoniae infection.

## 3 Conclusions

ACA is a cerebellar syndrome induced by autoimmune reactions. Studies have suggested that for patients with acute or subacute cerebellar syndromes of unknown etiology, anti-neurologic antibody testing should be performed through simultaneous testing of serum and cerebrospinal fluid specimens. Positive serum and (or) cerebrospinal fluid anti-neurologic antibodies may facilitate the diagnosis. In this case, a child with mycoplasma pneumoniae infection presented with acute cerebellar ataxia during the recovery of respiratory illness, and anti-CARP VIII antibodies in serum were positive. Following anti-immunotherapy, the symptoms resolved, and serum anti-CARP VIII antibodies became negative. The pathogenesis of this ACA is likely immune-mediated, triggered by a mycoplasma pneumoniae infection. However, literature has associated anti-CARP VIII antibodies with PCD, particularly in conditions such as melanoma, breast cancer, and ovarian cancer. Thus, in this case, anti-CARP VIII antibodies may serve as bystander markers of infection and contribute to cerebellar ataxia, although the exact pathogenesis remains unclear. However, there are some limitations to this case: no repeated MRI scans were performed, no CARP VIII antibody were detected in CSF analysis, and no biopsy was performed. Moreover, cases of CARP VIII antibody-mediated cerebellar ataxia are extremely rare, with no reported occurrences in children.

## Data Availability

The datasets presented in this article are not readily available because of ethical and privacy restrictions. Requests to access the datasets should be directed to the corresponding author.
